# The application of artificial intelligence in the intersection of metabolic dysfunction-associated steatotic liver disease and cardiovascular diseases

**DOI:** 10.3389/fimmu.2026.1788249

**Published:** 2026-04-01

**Authors:** Yu Kong, Hualei Chen, Yun Chen, Chengji Wang

**Affiliations:** 1 Department of Cardiology, Affiliated Hospital of Jining Medical University, Jining Medical University, Jining, Shandong, China; 2 Department of Gastroenterology, Jining No. 1 People’s Hospital, Jining, Shandong, China; 3 Department of Gastroenterology, Affiliated Hospital of Jining Medical University, Jining Medical University, Jining, Shandong, China

**Keywords:** artificial intelligence, cardiovascular disease, machine learning, metabolic dysfunction−associated steatotic liver disease, risk assessment

## Abstract

There is high comorbidity and complex pathological mechanisms between metabolic dysfunction−associated steatotic liver disease (MASLD) and cardiovascular disease (CVD), and the accuracy of traditional risk assessment tools is insufficient. The paper highlights that artificial intelligence (AI) including machine learning and deep learning capable of integrating clinical, imaging, and multi-omics data to enhance the precision of diagnosing MASLD and staging liver fibrosis, the related model has AUC greater than 0.85, and moreover, AI can also accurately predict CVD risk of patients with MASLD, which related model has AUC greater than 0.8 and whose performance is better than traditional scoring systems. In the medical field, deep learning facilitates the quantification of liver fat, along with the evaluation of coronary plaque and screening for lesions across different organs. Multimodal AI has the potential to reveal novel mechanisms and biomarkers of diseases. In addition to these challenges which include data quality and model generalization, the paper also points to future directions such as federated learning. AI offers a fresh perspective on assessing risks, understanding mechanisms, and implementing clinical interventions for MASLD-CVD.

## 1 Introduction

### 1.1 The intersection of MASLD and CVD: global burden and clinical significance

Metabolic dysfunction−associated steatotic liver disease (MASLD) is a chronic liver disease driven by metabolic dysfunction characterized by hepatocellular steatosis. MASLD has become the most common chronic liver disease worldwide, affecting 30-40% of the adult population (1). MASLD has changed from the original “non-alcoholic fatty liver disease” (NAFLD) to “metabolic-associated fatty liver disease” (MAFLD) to the “MASLD” determined by the international consensus in 2023, and the core of this series of changes is to improve the exclusionary diagnosis based solely on “non-alcoholic” to the positive diagnosis with “metabolic dysfunction” as the core, highlighting the central position of metabolic dysfunction in the occurrence and development of the disease (2, 3). The diagnosis of MASLD is based on hepatic steatosis and does not rule out coexistence with other liver diseases (e.g., alcoholic liver disease, viral hepatitis, etc.), supporting the “dual cause” diagnostic model (3). At the same time, specific cardiometabolic risk factors (such as overweight/obesity, type 2 diabetes, dyslipidemia, etc.) are added to make the diagnostic criteria more detailed and operable (4, 5). MASLD has a deep bidirectional relationship with cardiovascular disease (CVD). For MASLD patients, CVD is the main cause of death (6). Compared with patients without this disease, people with MASLD are more likely to have atherosclerosis, hypertension, myocardial infarction, stroke and other major adverse cardiovascular events (MACE) (7, 8). A latest review emphasizes that RWE has fully confirmed that MASLD is an independent risk factor for CVD, and this association remains consistent across real-world datasets from different regions and populations. For example, a real-world study of 18 million people in Europe showed that the risk of acute myocardial infarction in MASLD patients was 20% higher than that in non-MASLD populations (HR = 1.2, 95%CI: 1.1-1.3), while a study of 6.8 million people in Japan reported that the HR for cardiovascular disease in MASLD patients reached 2.6 (95%CI: 2.4-2.8) (9). This means that MASLD is an independent cardiovascular disease factor. This confirms an important need in the fields of liver disease and cardiology - the urgent need to establish more effective high-risk identification systems and develop scientific methods to improve outcomes.

### 1.2 The role of AI: a new paradigm for dissecting complexity

MASLD and CVD are not simply adhesive, but share a series of driving factors and interact with each other through multiple mechanisms (10). For patients with MASLD, commonly used universal cardiovascular risk assessment tools such as the Framingham Risk Score (FRS), Pooled Cohort Equation (PCE), Systemic Coronary Risk Assessment 2 (SCORE2), and the American Heart Association’s PREVENT equation may not have insufficient predictive accuracy (7, 11). These scoring tools are not only suitable for a narrow range of populations and rely only on limited linear variables, so relying solely on these traditional assessment methods is likely to underestimate the true cardiovascular risk in the metabolic-associated fatty liver disease (MASLD) population, thereby resulting in missed judgments in high-risk patients and lost opportunities for intervention (7). Artificial intelligence (AI) and its sub-fields, machine learning (ML) and deep learning (DL), provide a new paradigm with great potential for solving such complex problems. Compared with traditional statistical models, the unique advantage of AI is that they can accurately identify hidden high-order association patterns in large-scale and structurally heterogeneous datasets (12, 13). This is one of the important driving forces in the research and discussion of this review, enabling AI to deeply analyze the complex connections between clinical records, medical images, and multi omics data, and provide more accurate and personalized risk prediction solutions.

### 1.3 Overview of review content and literature sources

The relevant literature of the review was retrieved from PubMed, Embase, and Web of Science databases up to December 30, 2025, with the search strategy formulated as: (Artificial Intelligence OR Deep Learning OR Machine Learning) AND (Metabolic Dysfunction-Associated Steatotic Liver Disease OR Non-alcoholic Fatty Liver Disease OR metabolic-associated fatty liver disease) AND (Cardiovascular Diseases OR Major adverse cardiac events OR Coronary Artery Disease OR Coronary atherosclerotic cardiopathy OR Heart Failure OR Atherosclerosis OR Myocardial Infarction). Included studies were focused on AI-related applications in the intersection of MASLD and CVD. All types of studies were included except conference abstracts, unpublished research, letters, comments, and duplicate publications.

As this is a narrative review, our research team systematically classified and hierarchically sorted out the relevant literature by research topic and evidence type, mainly describing MASLD-CVD pathophysiological mechanism, AI for clinical risk stratification, AI for medical imaging analysis, multimodal AI for cross-organ screening, multi-omics AI for biomarker and mechanism exploration, and AI model challenges and future directions etc. Each category was further sorted by research design and evidence level, with classic findings, the latest progress and key conclusions extracted to form a hierarchical evidence system, providing solid and comprehensive literature support for the main content, analysis and conclusions of each review section.

## 2 The pathophysiological Nexus of MASLD and cardiovascular disease

There are profound and complex pathophysiological connections between MASLD and CVD, ranging from molecular to organ levels. The liver, as a core metabolic organ, plays an important role in interactions. MASLD is not a bystander, but the diseased liver actively promotes the development of cardiovascular risk through various means, forming a vicious cycle. The essence of this relationship is the prerequisite for building models using AI.

Insulin resistance (IR) is a common pathophysiological basis for mediating MASLD, type 2 diabetes and cardiovascular disease (CVD) (14). IR makes peripheral tissues such as muscles and fat less sensitive to insulin, leading to compensatory hyperinsulinemia. This systemic metabolic disorder can significantly affect the liver through multiple synergistic effects, promoting and exacerbating hepatic steatosis. Fat accumulation in the liver is a hallmark feature of MASLD and is also the initiator of a series of cascade pathological reactions that far exceed the live. Excessive accumulation of lipids in liver cells can trigger cellular stress and lipid toxicity, thereby inducing low-grade chronic inflammatory responses (15). Stressed hepatocytes and innate immune cells release large amounts of pro-inflammatory cytokines such as interleukin-6 (IL-6) and tumor necrosis factor-α (TNF-α) into the systemic circulation (10). This systemic inflammation induces vascular endothelial dysfunction and promotes the recruitment of immune cells into the arterial wall, which is key to the early stage of atherosclerotic plaques (10). At the same time, mitochondrial dysfunction in steatosis hepatocytes will increase the production of ROS and cause oxidative stress reaction, which will not only cause liver damage, but also may induce systemic oxidative stress, causing vascular endothelial damage and promoting the development of atherosclerosis (16). Atherosclerotic dyslipidemia is the main mechanism of coronary artery disease in MASLD patients. Dysregulation of liver lipid metabolism can cause specific dyslipidemia, manifested as hypertriglyceridemia, decreased HDL cholesterol, and significantly increased LDL ratio (10). This rare lipid feature has an abnormal atherogenic effect, which leads to atherosclerosis (16).

CVD and MASLD share a common genetic origin, with CVD being more common in MASLD. Specific genetic variations are associated with higher risk of liver fat and fibrosis progression. Let’s take the polymorphism in the papain like phospholipase domain protein 3 (PNPLA3) and transmembrane 6 superfamily member 2 (TM6SF2) genes as an example. These genetic factors affect the risk of liver and cardiovascular diseases by adjusting the distribution of circulating lipids, which well demonstrates the profound biological connection between liver fat metabolism and the cardiovascular system (15). For those more common molecular pathways, personalized treatment may arise (17, 18).

**Figure 1:**
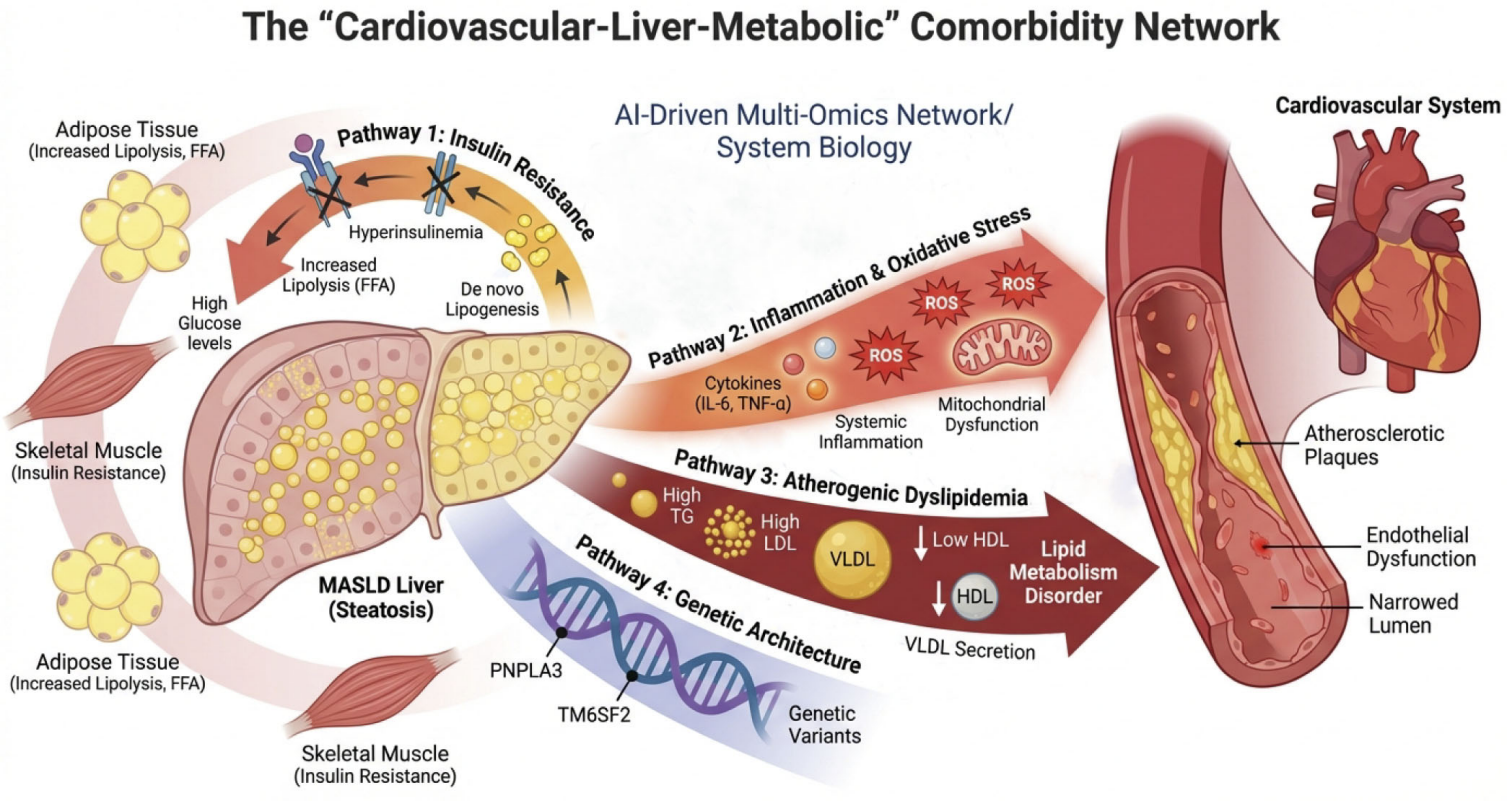
The main pathophysiological nexus between MASLD and CVD. This diagram clearly illustrates the “liver-cardiovascular-metabolic” interaction regulatory network driven by four core mechanisms. IR enhances adipose tissue lipolysis by promoting *de novo* adipogenesis in the liver, induces hepatic steatosis, and simultaneously triggers hyperinsulinemia and glucose metabolism disorders, thereby leading to systemic metabolic dysfunction. Chronic inflammation (represented by pro-inflammatory factors such as IL-6 and TNF-α) and oxidative stress (ROS originate from steatosis hepatocytes mediated by cellular stress and mitochondrial dysfunction), and both jointly damage the structure and function of vascular endothelium. Atherosclerotic dyslipidemia (manifested as high TG, high LDL, and low HDL) is directly caused by lipid metabolism disorders in the liver and can directly promote the formation and progression of atherosclerotic plaques. The shared genetic background (such as polymorphisms of genes like PNPLA3, TM6SF2, etc.) regulates the lipid metabolism process in the liver and jointly constitutes the genetic susceptibility basis for the onset of MASLD and CVD. The multi-system interaction network formed by the above mechanism provides an important theoretical basis for AI-driven mechanism modeling and therapeutic target screening. MASLD, Metabolic-associated fatty liver disease; CVD, Cardiovascular disease; IR, Insulin resistance; ROS, Reactive oxygen species; TG, Manifested as high triglycerides; LDL, Low-density lipoprotein; HDL, High-density lipoprotein.

MASLD and CVD have a variety of interlaced pathophysiological paths, such as insulin resistance, chronic inflammatory reaction, atherosclerotic dyslipidemia and genetic linkage, which together form a complex “cardiovascular liver metabolism” co-disease network (**Figure 1**). In this regard, only the overall perspective of system biology can be relied on to carry out analysis, and the multi group analysis technology driven by AI is an effective way to meet this demand.

## 3 AI for risk stratification using clinical and laboratory data

The most basic use of AI in the field of MASLD-CVD is to utilize existing clinical and laboratory data to construct predictive models, with the aim of using machine learning algorithms to model complex nonlinear relationships and applying them to statistical data of routine patients to improve the accuracy of diagnosis and CVD risk stratification. Thus overcoming the traditional risk scoring system.

### 3.1 Machine learning for MASLD diagnosis and fibrosis staging

The most critical step in MASLD risk management is to correctly identify the condition and assess its severity, with the most critical being liver fibrosis grading. Liver fibrosis is the strongest predictor of liver related mortality and all-cause mortality (19). For healthcare professionals, machine learning algorithms are a very valuable tool: they can identify important physical measurement indicators related to fatty liver disease (20). The combination of bioinformatics and machine learning techniques allows researchers to extract relevant genes and information for diagnosis and classification from gene expression data (21). The diagnostic performance of the algorithm trained by multiple types of indicators is better than that of existing non-invasive indicators (22). They also use cutting-edge technologies such as natural language processing to mine and analyze unstructured clinical records and imaging reports, thereby identifying some MASLD cases that have been missed by the International Classification of Diseases (23).

ML is also of great significance in evaluating the severity of diseases and determining the stage of liver fibrosis. Compared to traditional serological scoring systems, MASLD fibrosis score (MFS) has better diagnostic efficacy for advanced fibrosis (24). Hidden Markov models and other time series models can dynamically stratify fibrosis risk and provide early warning (25). Unsupervised ML can classify MASLD patients into different clinical subtypes, which have different prognostic outcomes. Deep learning models can distinguish the pathological stages of diseases and verify the relationship between characteristic genes and hepatocellular carcinoma (26, 27). Through the analysis of the genetic data set, ML can also find the common diagnostic core genes with complications such as MASLD and atherosclerosis (28). It provides a key support for the cross-study of MASLD and cardiovascular disease.

### 3.2 Predicting cardiovascular events in MASLD patients with machine learning

In recent years, significant progress has been made in the diagnostic modeling of MASLD, which has been strongly supported by multi-center clinical trials. With the establishment of a diagnostic model for MASLD, the next step is to predict the long-term cardiovascular event risk of this patient. Currently, long-term adverse cardiovascular events in internal medicine have become one of the key factors determining the prognosis of MASLD, and predicting their cardiovascular risk has become the most important clinical requirement (29). The application of machine learning can stratify the risk of MASLD patients and accurately predict their risk of developing CVD by analyzing various clinical data. ML has identified which features can effectively distinguish the long-term cardiovascular risk of MASLD patients. A study used a multi factor logistic regression classifier to construct a risk identification model for screening MASLD patients with high risk of experiencing long-term cardiovascular events in the future. The model demonstrated excellent predictive ability with an AUC of 0.86 (30). Another study turned to the application of gradient enhancement model on the MASLD cohort, and the AUC on the validation set was 0.817, indicating that it could well classify the risk of atherosclerotic cardiovascular disease (13). There are now many studies indicating that a range of machine learning algorithms can be used to effectively predict CVD risk in MASLD patients. These models are very effective in predicting outcomes such as major adverse cardiovascular events (MACE) and atherosclerotic cardiovascular diseases (ASCVD), and most of the AUCs reported are greater than 0.8 (12, 31–34). The application of ML has expanded to the CVD risk stratification of key populations, especially diabetes patients. A noteworthy example is the ability to accurately identify high-risk MASLD individuals in this queue, where the model showed AUC ranging from 0.81 to 0.84 (35). Emerging research is using ML fusion of multiple omics data to analyze gut microbiota and plasma metabolites together to identify MASLD patients at risk of cardiovascular disease. Changes in certain microorganisms (such as fecal bacteria, Ruminococcus) and certain metabolites (citral) are associated with CVD risk. A novel model combining these biomarkers with clinical data can detect high-risk individuals at a very early stage and intervene before pathological damage appears (36). The specific research results are shown in **Table 1**.

**Table 1:** Studies of predicting cardiovascular events in MASLD patients with AI.

Author	Patient cohort	AI model	Input data modality	Endpoint	Key performance (AUC)	Top predictors identified (via XAI)	Comparison models
Drożdż 2022 (30)	Single-center study 191 MASLD patients 47 CVD patients	LR	Biochemical parameters Demographical parameters Clinical parameters Carotid ultrasound parameters Arterial stiffness-related parameters Liver elastography parameters	CVD	Top-15 model: 0.87 (95% CI: 0.82-0.92)	Hypercholesterolemia, Plaque score L, Plaque score R, Duration of T2DM, Plaque area R, Age, Plaque area L, Betabloker, T2DM, Hypertension, cfPWV, ALT, eGFR, HbA1c, Obesity	ACC/AHA
Sharma 2022 (33)	Multi-center study: UK Biobank 846 MASLD patients 400 CVD patients	Single-domain models: RF, Lasso, Ridge, Naive Bayes, SVM, Logistic Regression, Neural Network Integrated model: Naive Bayes	Clinical Data Lifestyle Data Genetic Data	CVD	Single-domain mode (RF): Clinical data: 0.799 (95% CI: 0.779-0.817) Lifestyle data: 0.652 (95% CI: 0.639-0.665) Genetic data: 0.617 (95% CI: 0.599-0.637) Integrated mode (Naive Bayes ensemble)l: Clinical + Genetic: 0.820 (95% CI: 0.811-0.828) Clinical + Genetic + Lifestyle: 0.849 (95% CI: 0.840-0.855)	Age, Hypertension, Waist circumference, Time spent watching TV, IL16 gene mutations	NA
Li 2024 (36)	Single-center study Normal control (CTR): 61 Simple MASLD: 59 MASLD with CVD: 48 MASLD with CAD: 28	Random forest (RF)	Gut microbiota data Plasma metabolite data Clinical data	Stratifying CVD risk in MASLD patients	Metabolites + clinical parameters model: AUC: 0.90	Clinical Predictors: Age, Carotid intima-media thickness (IMT), Waist circumference/BMI, Blood lipids, Blood pressure Lifestyle-related Predictor: Xenobiotics/food additives Metabolomic Predictors: Citral, Biotin l-sulfoxide, Lithocholic acid taurine conjugate	NA
Li 2025 (13)	Single-center study 500 MASLD patients 434 CVD patients	RF, LR, GB, AdaBoost, XGBoost, LightGBM	Basic patient information, Laboratory parameters, Composite metabolic indicators	ASCVD	Optimal model: GB Training set: 0.918 (95% CI:0.890-0.944) Validation set: 0.817 (95% CI:0.739-0.883)	CHG index, CRI-II, Lp(a), Scr, UA, AST, SI, Sex.	China - PAR risk score
Shibata 2024 (12)	Single-center study 2962 MASLD patients 170 CVD patients	CART	Basic information, MASLD-related risk factors, Clinical and laboratory indicators	MACE	0.61-0.66	Age ≥60 years and ≥4 cardiometabolic risk factors	FRS, SCORE2-OP, ASCVD Risk Score
Dong 2025 (34)	Single-center study 2962 MASLD patients 170 CVD patients	RF, GLM, SVN	Clinical data, Immune inflammatory indicators	CHD	0.834(95% CI: 0.795-0.873)	NHR (top, OR = 1.375), NLR, SII, SIRI, NMR (risk), PNR (protective); age, T2DM, LDL-C, smoking, HT, TG.	NA
Liang 2025 (31)	Single-center study 7102 T2DM-MASLD patients 140 CAD patients	RF, SVM, XGBoost, LR	Clinical data, Radiomics data	CAD-RADS Grade 4 or Grade 5	Optimal model: XGBoost Training set: 0.883 (95% CI:0.779-0.943) Validation set: 0.852 (95% CI:0.792-0.912)	EAT/PAT radiomics features (MeshVolume, Entropy, etc.), clinical factors (diabetes duration, GLS, LDL-C, HbA1c).	NA
Nabrdalik 2023 (35)	Single-center study 1900 DM-NASLD patients	MLR	Basic information, clinical diseases, laboratory indicators	HF	Training set: 0.78 (95% CI: 0.75-0.81) Validation set: 0.67 (95% CI: 0.58-0.76)	AF, hyperuricemia, eGFR)	NA
Tang 2025 (32)	NHANES 674 MASLD patients 70 CVD patients	LR, SVM, KNN, RF, GBM, XGBoost, LightGBM, AdaBoost, CatBoost, Neural Network	Basic information, clinical diseases, laboratory indicators, imaging indicators	CVD	Optimal model: SVM 0.773	statin use, age, sex, diabetes, ALT, TyG-BMI	NA
Zhang 2025 (41)	Single-center 1073 MASLD patients 131 CAD patients	ResNet18	Tongue images, clinical features	CAD	0.933 (95% CI: 0.855–0.986)	Alkaline phosphatase, lymphocyte count, hematocrit, age, gender, smoking history, hypertension, diabetes	NA

XAI, Explainable artificial intelligence; CVD, Cardiovascular disease; ASCVD, Atherosclerotic cardiovascular disease; MACE, Major adverse cardiac events; CHD, Coronary atherosclerotic cardiopathy; HF, Heart failure; CAD, Coronary artery disease.

### 3.3 Beyond traditional risk scores: comparing AI models to conventional tools

Traditional CVD risk scores, such as Framingham risk score and ASCVD risk score, perform well in the general population, but it is still quite difficult to apply them to MASLD patients. For patients with MASLD, the development of CVD is not only due to metabolic problems, but also to actively worsen atherosclerosis, which is achieved through direct means such as chronic systemic inflammation and increased oxidative stress (37, 38) The traditional scoring system generally does not regard MASLD itself or its severity (like liver fibrosis) as an independent risk factors (39). The second point is that traditional methods mostly rely on cross-sectional or single time point data, which makes it difficult to grasp how risks change over time (25). The third part discusses that traditional tools may lack sufficient sensitivity to predict subclinical cardiovascular disease conditions (such as carotid plaques) (40). AI/ML models have shown significant advantages in predicting MASLD cardiovascular risk by fusing multidimensional nonlinear data and relying on algorithms to automatically identify complex patterns. More and more evidence suggests that ML provides a more accurate tool for predicting cardiovascular outcomes in MASLD compared to traditional methods. This study has confirmed its advantages, such as using metrics such as area under the curve (AUC), sensitivity, and specificity to measure its performance superiority. When comparing different models, it was found that the machine learning calculator was superior to the traditional ACC/AHA mixed queue equation. This study demonstrates a new tool for more accurate identification of cardiovascular events (30). At present, many studies have shown that it is very important for AI models used to predict CVD in MASLD patients to consistently achieve AUC values>0.8, as it represents an important improvement compared to previous risk scores, especially for patient populations that were previously not good enough (13, 36, 41) (**Table 1**). From an information perspective, there are relatively few models constructed using traditional variables, but AI can integrate ultrasound (42), coronary artery calcium (CAC) scans (43), tongue patterns (41), temporal trajectories (44) as well as some omics data such as metabolomics/transcriptomics data (including Metabolomics/Transcriptomics) (36, 45), AI models established through longitudinal data, such as hidden Markov models, can dynamically assess how liver fibrosis and cardiovascular risk develop. Traditional tools can only provide a one-time static assessment (25). A recent state-of-the-art review synthesizes the latest evidence linking MASLD to cardiovascular outcomes and discusses emerging therapeutic strategies targeting the cardio-hepatic axis. This review also notes that non-invasive diagnostics (including elastography, magnetic resonance elastography, and MRI-derived proton density fat fraction) and ML-based tools are improving MASLD staging and risk assessment. Its advantages include higher accuracy, earlier detection time, the ability to integrate complex multimodal data, and interpretive insights. But real-world implementation is variable and the cost-effectiveness of cardiovascular screening is underexplored (46).

### 3.4 The importance of explainable AI: identifying key clinical predictors

The value of explainable artificial intelligence (XAI) lies not only in its high accuracy, but also in its ability to “open the black box” and let clinical doctors know the specific reasoning behind each prediction. XAI techniques such as SHAP analysis and partial dependency graphs provide clinicians with intuitive insights into which clinical features drive artificial intelligence’s cardiovascular risk estimation, and understand the complex nonlinear relationship between these features and the final risk assessment (47, 48). Moreover, XAI can identify the most relevant predictive factors from a large amount of different clinical data, conduct research on specific outcomes (liver fibrosis, high-risk fatty hepatitis), and reveal potential causes (49). The XAI model provides personalized risk analysis, helping clinical doctors to distinguish between true positives, false positives, or negatives with clearer criteria, thereby optimizing screening and referral decisions (47). Research shows that in the context of MASLD, the most robust predictor of CVD may not be a single traditional factor. The predictive ability seems to lie more in the comprehensive biomarkers that capture the main disease mechanisms behind systemic inflammation and metabolic disorders. SHAP analysis has always shown that some comprehensive indicators, such as NHR, SII and fat sugar index, are as important in predicting CVD in MASLD as classic factors such as age and diabetes. This repeated proof highlights their importance (30). Through empirical verification, the AI model enhanced by XAI not only confirms systemic inflammation (caused by liver dysfunction) as the first predictive factor for cardiovascular risk, but also enhances its credibility by explaining this link and provides a deeper and more actionable.

## 4 Deep learning and the power of medical imaging

Although machine learning models trained on structured clinical data have proven their value, applying deep learning techniques to medical imaging has given new possibilities for non-invasive evaluation and risk prediction. Take Convolutional Neural Networks (CNNs) as an example, these deep learning models can automatically filter out complex and subtle visual features and patterns in images, even things that the human eye would overlook; And the insights they provide are quantitative and reproducible, making them an extremely valuable resource for studying liver disease and cardiovascular disease.

### 4.1 AI-enhanced hepatic imaging: quantifying steatosis and fibrosis

**Figure 2:**
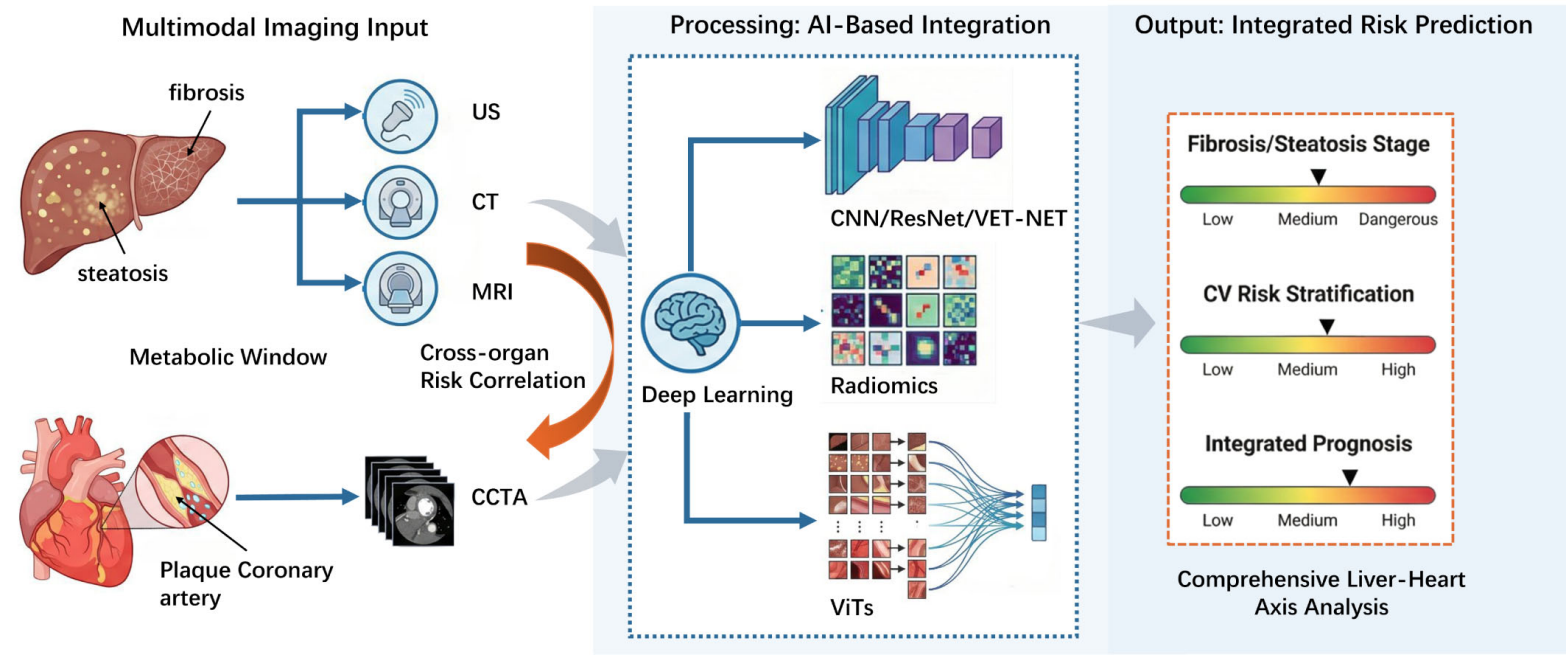
AI-based risk correlation and integration of cross-organ images. This diagram clearly illustrates the “liver-cardiovascular-metabolic” interaction regulatory network driven by four core mechanisms. IR enhances adipose tissue lipolysis by promoting *de novo* adipogenesis in the liver, induces hepatic steatosis, and simultaneously triggers hyperinsulinemia and glucose metabolism disorders, thereby leading to systemic metabolic dysfunction. Chronic inflammation (represented by pro-inflammatory factors such as IL-6 and TNF-α) and oxidative stress (ROS originate from steatosis hepatocytes mediated by cellular stress and mitochondrial dysfunction), and both jointly damage the structure and function of vascular endothelium. Atherosclerotic dyslipidemia (manifested as high TG, high LDL, and low HDL) is directly caused by lipid metabolism disorders in the liver and can directly promote the formation and progression of atherosclerotic plaques. The shared genetic background (such as polymorphisms of genes like PNPLA3, TM6SF2, etc.) regulates the lipid metabolism process in the liver and jointly constitutes the genetic susceptibility basis for the onset of MASLD and CVD. The multi-system interaction network formed by the above mechanism provides an important theoretical basis for AI-driven mechanism modeling and therapeutic target screening. MASLD, Metabolic-associated fatty liver disease; CVD, Cardiovascular disease; IR, Insulin resistance; ROS, Reactive oxygen species; TG, Manifested as high triglycerides; LDL, Low-density lipoprotein; HDL, High-density lipoprotein.

Artificial intelligence is quietly changing the way we diagnose liver disease, improving accuracy, speed, and reliability through automated scanning analysis, improving image quality, and identifying patterns that are invisible to the naked eye (50, 51) (**Figure 2**). Ultrasound examination is one of the most commonly used tools for screening fatty liver, and there may be some subjective factors when using ultrasound for liver disease detection. The AI assisted ultrasound diagnosis of liver diseases can effectively distinguish fatty liver from normal liver tissue using SVM classifier, and its results are consistent with the explanations given by doctors (52). Secondly, the deep learning index showed the best diagnostic ability in distinguishing moderate and severe MASLD (AUC = 0.958), indicating that deep learning has the highest sensitivity and specificity in evaluating the severity of MASLD (53). A large dataset annotated with liver biopsy results is used to train these robust artificial intelligence models. The combination of standardized imaging protocols and artificial intelligence tools may make ultrasound attenuation imaging more reproducible and clinically valuable (54). The most accurate method for measuring liver fat is the MRI technique called proton density fat fraction (PDFF), which is based on chemical shift encoding (CSE-MRI), which is currently the gold standard (55). Not all medical institutions can provide standard CSE-MRI sequences. The analysis using CNN shows that there is a high possibility of estimating PDFF directly from standard clinical T1 weighted IOP images. When compared with the reference standard CSE-MRI, not only is the consistency higher, but the measurement deviation is also smaller, paving the way for a wider range of clinical applications (56). The VET Net deep learning framework can efficiently and robustly handle measurement variations caused by different MRI scanners and acquisition parameters. The advantage of VET Net is its consistent performance in different clinical environments. Even when testing data from multiple centers, it showed minimal PDFF bias. The results of repeated measurements in different liver ROIs are comparable or even better than the standard reference method, demonstrating the reliability of this method regardless of the equipment or protocol used (57). CT is not the best choice for quantifying liver fat. However, using deep learning reconstruction techniques can improve image contrast and lesion visibility, and may allow for more accurate analysis of liver tissue features through CT scanning (58). By combining images from ultrasound, CT, MRI, and elastography to create artificial intelligence models, this approach provides a comprehensive and non-invasive method for staging liver fibrosis, greatly reducing clinical reliance on invasive liver biopsies (50, 51). The ordinary CT scan image analysis model based on ResNet has a very high diagnostic accuracy for non-invasive liver fibrosis. It achieves an AUC of 0.9-0.97 at different fibrosis thresholds (≥ F1–F4), especially in identifying late stages, providing clinical doctors with a practical and cost-effective auxiliary tool (59). The TMM integrated CVD system combines radiomics and advanced deep learning techniques to achieve precise METAVIRV stage classification on MRI. In animal model experiments, its overall accuracy reached 72.14%, which is better than current mainstream methods and proves the possibility of using radiomics combined with deep learning for high-precision staging (60). The reproducibility of imaging-based AI models across scanners, vendors, and acquisition protocols remains insufficiently validated. Differences in image quality, reconstruction parameters and site-specific workflows may affect feature stability and model performance. Although deep learning frameworks such as VET Net can enhance the robustness of models when used across platforms, Future studies still should place greater emphasis on multi-center external validation, protocol harmonization, and robustness testing across platforms before broad clinical deployment.

### 4.2 AI in cardiovascular imaging: plaque characterization and risk assessment

Artificial intelligence is changing the field of cardiovascular imaging, especially in the analysis of coronary artery plaques and patient risk assessment, demonstrating great potential and practicality (61, 62). Coronary CT angiography (CCTA) is currently the main non-invasive method for evaluating coronary artery plaques. Artificial intelligence has greatly improved the in-depth analysis and efficiency of plaques by utilizing advanced algorithms (63). On the one hand, using deep learning algorithms can automatically and quickly segment the entire coronary artery tree from CCTA images. Thus, precise quantification of total plaque volume, volume of different components (calcification, fibrous tissue, and lipid rich necrotic core), and plaque burden information can be obtained (63–65). It surpasses the traditional manual measurement and observer distinction, obtaining objective and reproducible quantitative results (63). On the other hand, artificial intelligence models can utilize morphological features (such as positive remodeling, punctate calcification, low-density plaques) and structural features to identify high-risk vulnerable plaques that are prone to acute cardiovascular events (63, 66). It breaks through the traditional model of assessing risk solely based on the degree of luminal stenosis and facilitates early intervention measures for diseases (63). Moreover, artificial intelligence can analyze subsequent CCTA scans to detect even small changes in plaque volume and composition, providing clinical doctors with subjective and accurate information to evaluate the effectiveness of patients’ enhanced lipid-lowering therapy or targeted drugs (63). In fact, the power of artificial intelligence lies not only in simplifying workflows. It can link imaging data with the clinical condition of patients, creating more reliable risk prediction models. These integrated models are always much better than traditional scoring systems and have excellent accuracy, specificity, and sensitivity (67). The use of deep learning in large-scale studies can not only automate the measurement of coronary artery plaques, but also develop personalized risk thresholds for each patient. This provides doctors with an intuitive and personalized method to stratify cardiovascular risk (65) (**Figure 2**).

### 4.3 Opportunistic screening: leveraging AI to detect cross-organ pathology

In the era of precision medicine, tools driven by artificial intelligence have taken risk assessment one step forward. By integrating data from multiple anatomical structures, they can display the pathophysiological connections between different organs. The focus will shift from evaluating individual organs to a holistic and multi organ perspective, providing more comprehensive risk profiles and warnings (68). To achieve this, this tool relies on automatic segmentation technology to first obtain high-quality imaging data of various organs, then merges these multi organ data together to identify key features from each source, and finally uses advanced machine learning algorithms to create predictive models that can display the hidden complex nonlinear relationships in these integrated data (69–71). Regard the liver as the ‘metabolic window’ for cardiovascular health. The imaging data includes subtle metabolic and vascular changes, which can serve as early risk markers for heart disease (72). Deep learning models are capable of autonomously learning and extracting patterns from complex data that are invisible to the human eye, especially the ability of vision converter models to extract hidden information from massive liver MRI scans, transforming these intrahepatic images into quantitative risk estimation indicators for cardiovascular disease (72) (**Figure 2**). An astonishing study has shown this, as researchers use artificial intelligence algorithms to complete routine imaging work for coronary artery calcification (CAC) examination, teaching them the opportunity to calculate the degree of greasiness in the liver from the same batch of photos. Moreover, it has been found that the liver oil fraction generated by AI is indeed an independent factor for predicting heart disease onset, death, and other factors. Importantly, this method does not increase the cost or cause for patients. Radiation dose (43). Another study trained a deep learning model using single-layer liver MRI scans. The AUC of the main cardiovascular events predicted by this model reached 0.70, which proves its reliable predictive ability and practical possibility for risk grading (72). This study suggests that artificial intelligence can obtain very deep biological insights from liver imaging and can apply its foresight to other diseases. This study indicates that artificial intelligence is a truly exciting and important direction in the field of medical imaging. From a management standpoint, MASLD should be considered within a broader metabolic axis in which interactions between the heart and liver can influence disease progression. It further highlights that incretin-based therapy shows major benefits for both MASLD and cardiovascular disease, reinforcing the need for multidisciplinary and holistic approaches that address liver and heart health simultaneously (73).

### 4.4 Radiomics and deep learning architectures for integrated risk prediction

These advanced imaging applications are supported by radiomics and complex deep learning models. Radiology extracts large amounts of quantitative data from medical images and captures detailed information about tissue texture, shape, and density. In this process, traditional machine learning can use manually crafted features, but deep learning models such as convolutional neural networks (including ResNet or DenseNet architectures) can directly learn the most useful characteristics from raw pixels (74). Emerging new structures such as VisualTransformers (ViTs) have great potential for development in this task, as they are highly adept at identifying a wide range of spatial patterns and long-term dependencies contained within images. This makes them very suitable for dissecting complex anatomical structures, such as those found during liver MRI scans (72). This powerful architecture now empowers integrated models that can directly infer patient prognosis from medical images, greatly reducing the gap between scanned data and clinical prognosis. In addition to model performance, successful implementation will also depend on effective integration into routine clinical workflows. For real-world adoption, AI systems should provide timely, interpretable results that are readily accessible through PACS (Picture Archiving and Communication Systems) and EFR (electronic health record systems). Avoiding additional operational burden for clinicians will be critical to achieving sustained acceptance in daily practice (75, 76).

## 5 Uncovering novel mechanisms with multimodal and multi-omics AI

### 5.1 Multimodal data fusion: integrating clinical, imaging, and ‘omics’ data

**Figure 3:**
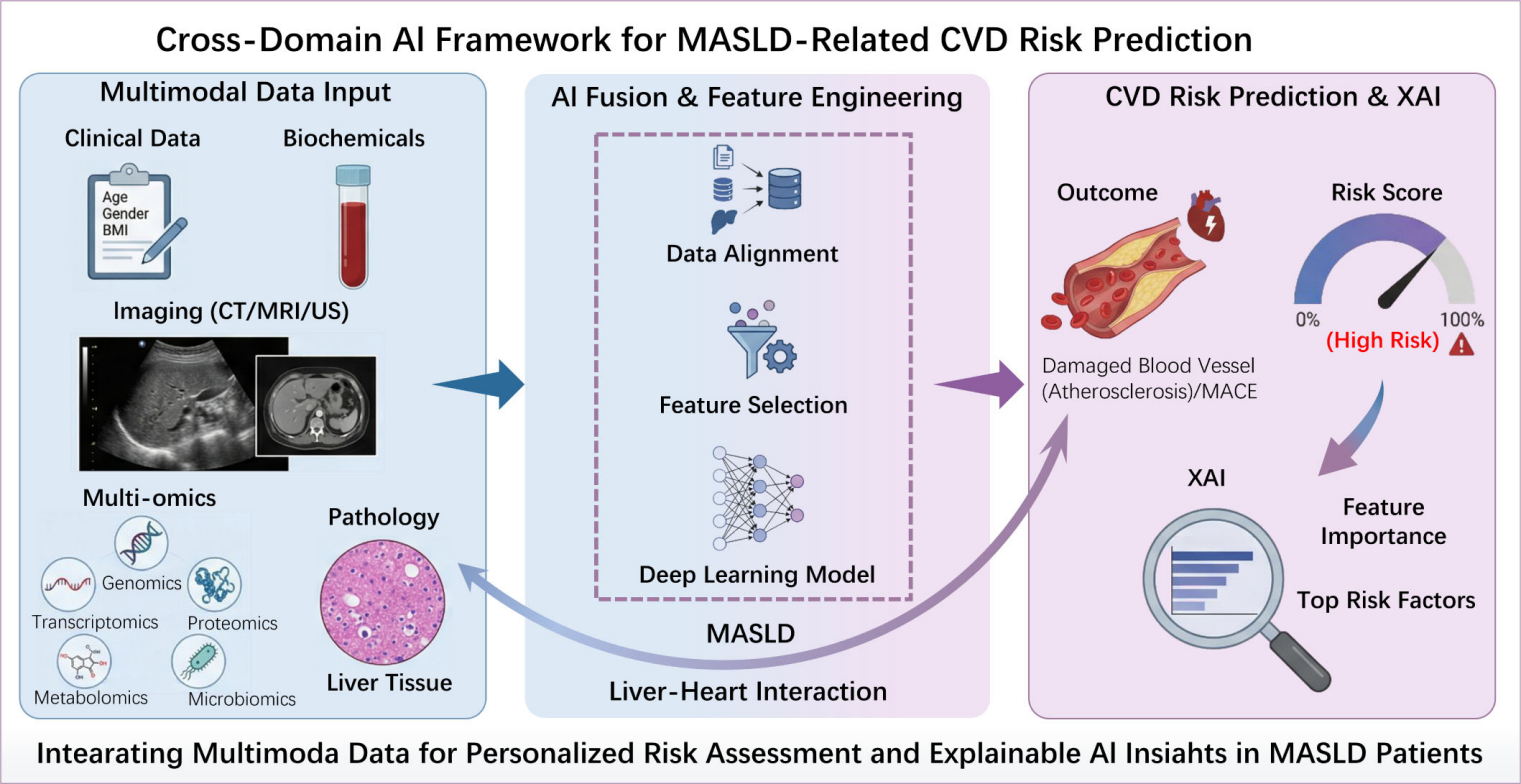
Framework diagram for predicting MASLD-CVD risk based on multimodal AI data fusion. This framework takes clinical data, imaging features, and multi-omics data as input layers, and after processing by the federated learning model, the feature fusion module extracts cross-dimensional correlation features, and finally outputs the CVD risk prediction probability. The XAI Interpretation Module ranks the weights of key predictive features, providing an interpretable, verifiable, and domain-compliant basis for model decisions. MASLD, Metabolic Associated Steatotic Liver Disease; CVD, Cardiovascular Disease; XAI, Explainable Artificial Intelligence; MACE, Major Adverse Cardiovascular Events; CT, Computed Tomography; MRI, Magnetic Resonance Imaging; US, Ultrasound.

The integration of multimodal artificial intelligence models with different data types provides a promising path for precision medicine, which can better predict the future cardiovascular event risk of MASLD patients. The combination of multimodal data refers to the integration of data from various sources and types (such as clinical information, imaging, pathology, and genomics) to capture multidimensional features of diseases. This method solves the problem of a single data source and greatly improves the predictive ability of artificial intelligence models (77, 78). So far, there has been no research using multimodal artificial intelligence models to predict the cardiovascular disease risk of MASLD patients. Significant progress has been made in utilizing multimodal artificial intelligence models for the diagnosis, prognosis, and risk stratification of other diseases. The technical framework, data integration strategy, and modeling methods developed by this research institute have important guiding significance for establishing a predictive model for cardiovascular risk in MASLD patients (77). For example, the research results of integrating retinal fundus images with clinical risk factors have developed an AI model that greatly improves CVD prediction. In the UK Biobank cohort, the AUC of this model reached 0.872 (79). In addition to the above, this multi-mode AI model has also achieved good results in cancer prognosis and recurrence risk prediction (80), non-invasive detection of cardiovascular parameters (81), diabetes management and control (82), and estimation of critical risk of pulmonary infectious diseases (83). These research results provide a clear direction for the use of multiple data to predict the risk of cardiovascular events in patients with MASLD. The theoretical framework for predicting the risk of CVD development in MASLD patients via multimodal AI data fusion is shown in **Figure 3**.

### 5.2 AI-driven ‘omics’ analysis: identifying novel biomarkers and causal pathways

The omics analysis driven by artificial intelligence has opened up a new path for predicting cardiovascular risk in MASLD patients. It provides scientific basis for accurate prediction and targeted intervention by identifying potential new biomarkers and elucidating key causal pathways of disease co-occurrence (84, 85). The multi omics analysis led by artificial intelligence integrates data from different biological layers (genomics, transcriptomics, proteomics, metabolomics, and microbiology) to form a more comprehensive disease landscape, which significantly improves the accuracy of prediction models from a comprehensive perspective (85).

Artificial intelligence driven omics analysis can critically identify various new biomarkers associated with MASLD and CVD, and proteins such as CNPY4 and ENTPD6 have been shown to have causal relationships with MASLD through Mendelian randomization. These biomarkers are now worth studying to understand their roles in the cardiovascular system (86). Zhang et al. screened HNF4A and LTBP4 as the core genes of non-obese MASLD and CAD comorbidities through a variety of ML algorithms, which were involved in the comorbidity process through cholesterol metabolism and TGF- signaling pathways and have good diagnostic value (87). For metabolic biomarkers, things of great interest include AMP, 13-HODE, and uric acid, as well as gut microbiota derived metabolites such as PAC and PAG. This disorder suggests the possible existence of systemic metabolic disorders (88–90). Taking the microbiota-gut-liver axis as the core framework, through the integration of multi-omics data and ML, the molecular characteristics of MASLD and the microbiota-gene interaction mechanism are systematically revealed, providing a core basis for the clinical application of the co-disease of MASLD and CVD (91). Gut microbes are central to the development of MASLD. Microbiome studies have identified unique microbial interaction modules and metabolic pathways specific to this disease. Two key pathways (heme synthesis pathway and thiamine metabolism pathway) have been confirmed to associate with oxidative stress damage and energy metabolism disorders in CVD (92). Screening the transcriptome of liver tissue or peripheral blood mononuclear cells can display characteristic gene expression, including genes such as CAPG and SPP1 associated with liver disease development. These gene expression changes often indicate potential systemic inflammation and endothelial dysfunction (93). Besides, activated glycolytic pathways in MASLD patients lead to abnormal immune cell infiltration in liver tissue, and this is a common pathological feature connecting MASLD and CVD (94). Artificial intelligence driven omics analysis can also elucidate the causal relationship between MASLD concentric vascular diseases. At a deeper level, AI driven pathway enrichment analysis can identify common biological mechanisms. The common pathways involved in these two diseases include insulin signaling, inflammatory response (including IL-6/JAK/STAT pathway, lipid metabolism, and oxidative stress), as well as purine metabolism and cellular energy sensing pathways such as AMPK signaling (23, 89, 95). With the help of artificial intelligence driven spatial genomics, we can now accurately identify abnormal interactions between specific types of liver cells, such as aging endothelial cells and hepatic stellate cells. The signaling molecules generated by these interactions, such as IGFBP7, may enter the bloodstream and directly affect vascular wall cells. More and more are referred to as the “hepatic heart” or “hepatic vascular” axis (23). Research has found that FMT can alter the methylation pattern of liver DNA in MASLD patients, thereby affecting genes such as TARS and ZFP57. AI can analyze how these epigenetic changes regulate gene expression in the liver and cardiovascular system, and help explain how environmental factors work through comorbidities and genetic interactions (88).

ML excels at processing large amounts of complex omics data and can accurately locate biomarker combinations that are often overlooked in traditional analysis. The current research has applied machine learning to the GEO database dataset to identify key genes related to MASLD, and prediction models based on these genes have strong diagnostic accuracy for the disease (96). It can also be used to predict the risk of cardiovascular events by training models to identify specific omics features that predict future cardiac complications, providing a shortcut for risk stratification. A recent study conducted complete whole genome, epigenome, and transcriptome sequencing on MASLD patients. After using SVM algorithm, they found a method framework that can effectively distinguish characteristic genes at different stages of the disease. The same framework may be able to screen specific “gene features” related to cardiovascular complications in MASLD patients, paving the way for a new blood diagnostic panel (93). In addition to risk stratification, artificial intelligence can also integrate different data sources to create personalized prediction models. In metabolic syndrome (MetS) research, artificial intelligence models have significantly improved the detection and prognosis of MetS and related diseases by integrating non-traditional biomarkers such as lipidomics and proteomics (97). Due to the close relationship between MASLD and metabolic syndrome, a comprehensive multi omics AI model can be effectively used to predict the risk of cardiovascular events, including myocardial infarction and stroke, in individual MASLD patients (98). The details of the application of driven omics analysis in MASLD and its association with CVD are shown in **Table 2**.

**Table 2:** The application of AI-driven omics analysis in MASLD and its association with CVD.

Study	Research topic	Data type	Key biomarker/pathways	Related to MASLD-CVD
Feng 2025 (86)	Identification of biomarkers for MASLD	Genomics Data Proteomics Data Clinical Data	APOE、CNPY4、ENTPD6、HLA-A、SCG3、TOR1AIP1 ALB, ApoA, GGT, HDL-C, IGF-1, UNa, STP, TG) STP	* Directly present the trend association between STP and cardiovascular mortality risk. * Using cardiovascular risk properties of MASLD-related clinical biomarkers (HDL-C, TG, IGF-1, GGT), suggest MASLD and cardiovascular diseases may associate via common metabolic pathways.
Zhang 2026 (87)	Explore the core genes of non-obese MASLD and CAD, and reveal the association mechanism between them	Clinical Data Genomics Data	HNF4A, LTBP4 Glucolipid metabolism regulation pathway LTBP4-TGF-β signaling pathway	* Cholesterol metabolism disorder is a key pathological mechanism for non-obese SLD complicated with CAD. * HNF4A and LTBP4 are core shared biomarkers of non-obese SLD (MASLD) and CAD. * High HNF4A, low LTBP4 link MASLD and CVD by regulating glycolipid metabolism and the TGF-β signaling pathway.
Gonçalves 2023 (88)	Reveal the role of the “gut microbiota- plasma metabolites - liver DNA methylation” cross-organ regulatory network in MASLD	Clinical Data Multi-omics Data Microbiome Data Metabolome Data Epigenetics Data	Phenylacetic acid-derived metabolites:PAC, PAG Choline-derived metabolites: palmitoylcholine, oleoylcholine Glycerolipid metabolism-related metabolites Liver DNA Methylation	* Key metabolites regulated by FMT (PAG, choline derivatives) are associated with cardiovascular diseases * FMT may indirectly affect cardiovascular health by improving metabolic disorders of MASLD
Sun 2025 (89)	Elucidate the molecular mechanisms of MASLD induced by environmental exposure and screen specific metabolic biomarkers	Basic Experimental Data Multi-omics Data Proteomics Data Metabolomics Data Clinical Data	AMP, 13-HODE, Hippuric Acid Dysregulation of PPAR Signaling Pathway, Abnormal Purine Metabolism	The PPAR signaling pathway-purine metabolism can regulate lipid metabolism, inflammatory response, and oxidative stress, and is also a key regulatory factor for CVD.
Oh 2024 (93)	Screen and validate a signature gene set for accurate discrimination of MASLD progression stages	Multi-omics Data Genomic Data Epigenomic Data Transcriptomic Data Clinical Data Basic Experimental Data Public Databases	CAPG、HYAL3、WIPI1、TREM2、SPP1、RNASE6	It does not directly explore the association with cardiovascular events, but provides important references for developing cross-disease biomarkers
Fujiwara 2025 (23)	Reveal the mechanism linking hepatic fiber morphological heterogeneity to MASLD progression	Clinical Data Multi-omics Data Transcriptomic Data Basic Experimental Data	FibroPC4, IGFBP-7 Senescent Endothelial Cell-Inflammation Pathway	It does not directly explore the association with cardiovascular events, but provides important references for developing cross-disease biomarkers
Zhang 2025 (96)	Screening and validating potential inflammation-related biomarkers for MASLD progression	Public Databases Multi-omics Data Epigenomic Data Transcriptomic Data Basic Experimental Data	UBD/FAT10、STMN2、LYZ、DUSP8、GPR88 * Inflammation-Related Pathways: NF-κB Signaling Pathway, Chemokine-Mediated Signaling Pathway * Metabolic Regulation Pathways: PPAR Signaling Pathway, Steroid Biosynthesis and Homeostasis Pathway * Fibrosis and Cell Damage Pathways: ECM-Receptor Interaction Pathway, p53 Signaling Pathway	It does not directly explore cardiovascular event association, but provides potential candidate genes for investigating “MASLD-cardiovascular disease comorbidity” molecular mechanisms
Hui 2025 (95)	Develop a non-invasive biomarker panel to distinguish the severity of MASLD	Public Database Multi-omics Data Transcriptomic Data Metabolomic Data Lipidomic Data Clinical Data	5-ALA, Mesaconic acid, Shikimic acid, PC O-35:3, PI 36:2 * Lipid Metabolism and Atherosclerosis-Related Pathways: AMPK (/PGC-1α/SREBP-1c) Pathway, PPARα Pathway, REBP-1c Pathway * Insulin Resistance and Endocrine Regulation Pathway * Inflammation and Oxidative Stress-Related Pathways: Nrf2 Pathway, TNF/IL-17 Pathway * Amino Acid and Nucleotide Metabolism Pathway	Potential Association Between MASLD and CVD: Sharing the Core Pathological Pathway of “Lipid Metabolism Disorder-Atherosclerosis”
Huneault 2024 (90)	Validate the diagnostic value of a modified screening model for pediatric MASLD	Clinical Data Metabolomic Data	Leucine/Isoleucine, Tryptophan, Serine, LysoPC 18:1, LysoPE 20:0, Dihydrothymine * Amino Acid Metabolism Pathway * Lipid Metabolism Pathway * Pyrimidine Metabolism Pathway * Glutathione Synthesis Pathway	Some metabolites (e.g., branched-chain amino acids linked to insulin resistance, lysophospholipids linked to lipid metabolism disorders) are associated with both MASLD and cardiovascular risk.
Nychas 2025 (92)	Exploration of Robust and Highly Specific Gut Microbiome Signatures in MASLD	Genomic Data Microbiome Data Metabolomic Data Clinical and Phenotypic Data	Eubacterium hallii, Blautia obeum, Flavonifractor plautii, Intestinibacter bartlettii, Dialister sp. CAG 357,etc. Protoheme, DOPAC, B1/Thiamine, Ile/I, Ade, 4-Hyp, D-TA, cUMP * Microbial Functional Pathways: SCFA Synthesis Pathways, Bile Acid Metabolism Pathway, Purine Metabolism Pathway, Lysine Biosynthesis Pathway * Host-Microbe Crosstalk Pathways: SCFA Synthesis → Propionate → Hepatic Lipid, Heme Synthesis → Protoheme → ALT, Branched-Chain Amino Acid Metabolism → L-Isoleucine → Insulin Resistance	The association of cardiovascular risk indicators with microbial markers of MASLD was analyzed by inclusion in the CVD cohort
Chen 2025 (94)	Investigation on the Identification of Glycolysis-Related Key Genes in MASLD and Their Association with Immune Infiltration	Multi-omics Data Transcriptomic Data Genomic Data Clinical Phenotype Data *In Vivo* Experimental Data	ALDH3A1, CDK1, DEPDC1, HKDC1, SOX9 M1 Macs, M2 Macs, rDCs, CD4+ mR T cells * Glycolysis/Gluconeogenesis pathway * M1 macrophage polarization pathways: TLR4/NF-κB pathway, IL-6/JAK/STAT3 pathway * CCL2-CCR2 pathway * Glycolipid metabolism disorder-related pathways * Transcription and epigenetic regulation pathways of key genes	* The mechanism of “glycolysis-driven M1 macrophage polarization” in MASLD also applies to CVD. * The abnormal expression key glycolytic genes participate in systemic metabolic regulation may indirectly increase CVD risk.
Comertpay 2025 (91)	Identification and Validation of the Diagnostic Value of Key Molecular Biomarkers for MASLD Centered on the “Microbiota-Gut-Liver Axis”	Public Databases Multi-omics Data Transcriptomic Data Genomic Data Microbiome Data Clinical Data	CLDN1, TJP2, TLR2/TLR4, MAPK14, HMOX1, PPARG, TJP2, EGFR, GSK3B let-7a-5p, miR-195-5p, miR-124-3p NFKB1, SP1 Akkermansia muciniphila, Lactobacillus rhamnosus GG, Faecalibacterium prausnitzii * TLR2/TLR4 Inflammatory Pathway	* MASLD highly overlaps with CVD risk factors and shares mechanisms of inflammation and metabolic abnormalities. * Key genes of MASLD (EGFR, TLR2/TLR4, PPARG) and regulatory pathways (NFKB1 inflammatory pathway) are also involved in CVD.

### 5.3 Systems biology approaches to model the MASLD-CVD interactome

Systems biology integrates multiple omics data from genomics to metagenomics, using tools such as network analysis to process this information. This approach transforms the existing epidemiological association between MASLD and CVD into a biological network and identifiable molecular mechanism (99–102). It displays the genetic background and core pathways shared by two diseases, including those related to inflammation, immune response, lipid metabolism, and coagulation, and identifies important communication nodes between different organs. It also identifies potential biomarkers and multiple target therapy methods (103–105). The future challenge is how to further develop these methods so that patients can be stratified more finely and ultimately achieve precision medicine (99). And it is necessary to transform the research results of systems biology into non-invasive methods that can be used for early clinical detection, risk prediction, and efficacy monitoring (102). Due to the continuous growth of multi omics data resources and the increasing complexity of computational models, as the interaction between MASLD and CVD becomes more complex, biologists of the system will also gain more new insights. This helps us develop better and more personalized prevention and treatment measures.

## 6 Challenges and future directions of AI in clinical application

### 6.1 Data quality, algorithmic bias and clinical translation of AI models

The performance of AI models is highly dependent on the quality and representativeness of training data, and current research is obviously insufficient in this regard. A key limitation is that the use of single-source, retrospective data may easily introduce bias and restrict the model’s generalization ability (12, 13, 106). Second is the dependency and clinical feasibility of feature selection. Some key predictive indicators are unconventional clinical examination items, which is not conducive to their promotion at the grassroots level (13, 106). The last problem is that there are differences in how to define its end point. This inconsistency makes it difficult to compare these models, and constrains their predictive role. The universality of this model is the main issue in its clinical translation. Most existing models have not been rigorously tested in various large-scale clinical environments (13, 107). The performance of the training set and the validation set is significantly different, indicating overfitting and untested real-world reliability (13). In addition, the high heterogeneity of MASLD, the data limitations of people from different ethnic regions, as well as the bias and black box characteristics of the algorithm itself, further weaken the clinical credibility of the model (6, 108, 109). Moreover, even with high-quality data, modeling algorithms will continue or amplify biases, and their ‘black box’ nature will further undermine trust in the clinical environment. There is currently no optimal algorithm that is suitable for all situations (12, 13, 40). The model lacks true explanatory power. Although tools such as SHAP can show how each feature contributes to the score, they only provide a final risk number rather than indicating potential biological pathways (13, 39, 40). On the basis of baseline data, static models cannot follow dynamic risks. Moreover, most of the models are static models based on baseline data, which cannot track the dynamic changes of MASLD and related risk factors, and it is difficult to accurately determine the real-time risk of patients (13, 40, 107).

The use of artificial intelligence models to predict CVD risk in MASLD patients has moved from concept to retrospective validation, demonstrating the potential of layered tools (12, 13, 40, 107). However, there are still many obstacles to overcome in order to apply them in the real world, such as data bias, overlooked patient differences, lack of prospective testing, difficulty in integrating with existing workflows, and unresolved ethical regulatory issues (6, 17, 110, 111). Perhaps there will be a breakthrough by conducting prospective intervention trials in prospective intervention research, creating dynamic multimodal models, and forming interdisciplinary and standardized evaluation and application ecosystems. This way, we can focus on improving outcomes rather than just estimating risks.

### 6.2 The future landscape: federated learning, generative AI, and integrated decision support

In the field of predicting the risk of MASLD in patients, federated learning, generative AI, and integrated decision support are promising future directions. However, these cutting-edge technologies are not fully covered in the current literature (12, 39, 45). Under the premise of protecting data privacy, federated learning can combine multi-institutional data training models to solve the limitations and data silos of single-center research. It can provide assistance for CVD risk prediction, which requires a large amount of multidimensional data (12). Now, in the research of artificial intelligence models, there has been a shift from traditional machine learning to more complex integrated systems. There is evidence to suggest that machine learning can effectively classify risks into different levels, and MASLD itself has significant predictive power (12, 39). In the future, we should apply federated learning to collaborative data analysis and use generative artificial intelligence to improve model performance. Our goal is to establish an integrated, multi-modal, interpretable system for early and accurate management of cardiovascular risk in MASLD patients.

### 6.3 Environmental sustainability of AI in routine clinical practice

Current literature has not systematically explored the environmental sustainability of AI at the intersection of MASLD and CVD. However, its clinical applications already show significant positive impacts. AI enables non-invasive diagnosis and precise stratified screening. These approaches reduce invasive procedures and overtreatment. Consequently, they lower the overall consumption of medical resources (12, 25). AI also facilitates dynamic monitoring and personalized treatment. These capabilities delay disease progression and help avoid resource-intensive and high-energy treatment stages. Besides, AI reduces the environmental impact associated with ineffective medications (112). It can also integrate multi-source data, mine low-cost biomarkers, improve the efficiency of medical services, and promote the popularization of grassroots risk monitoring (39). This technological synergy will make its potential even more pronounced. Ultimately, AI will play a key role in advancing environmentally friendly healthcare.

## 7 Conclusion

The fusion of metabolic dysfunction related fatty liver disease and cardiovascular disease is one of the most serious public health threats of our time. However, the complexity and multifactorial nature of their common pathophysiological basis highlight the shortcomings of traditional risk assessment tools, and clearly require more complex analytical methods to address them. Artificial intelligence provides unprecedented opportunities to address this challenge, starting to unravel this complexity, improve risk stratification, and uncover new biological phenomena.

This review traces back to the development of artificial consciousness in this field. Starting from machine learning methods that can predict clinical risks, to the emergence of powerful applications of deep learning in medical images, which have opened up new paths for opportunistic screening, the new limit of this research is multimodal and multi omics artificial intelligence. It is now able to identify clues that connect the liver and heart, and shift from previous predictions to more in-depth cause analysis work. The clinical characteristics of patients and non-imaging biomarkers such as blood are convenient and low-cost, and are suitable for grassroots development. Lipids, inflammatory factors, plasma metabolites, etc. have been confirmed to be effective indicators for the diagnosis of MASLD and the risk prediction of CVD in AI models. Incorporating non-imaging indicators into the training of AI models can reduce the reliance on specialized imaging examinations such as MRI and CCTA, thereby enhancing the practicality and applicability of these models in primary care settings. By combining the clinical, metabolomics, and microbiomics biomarkers verified in this study, the combined use of artificial intelligence and non-imaging diagnostic methods can compensate for the limitations of medical imaging in terms of accessibility and routine application. Meanwhile, the fusion of image and non-image data is the key to achieving hierarchical management of MASLD CVD. Through multimodal AI, the initial screening risk stratification and dynamic follow-up assessment of high-risk groups can be completed, improving the level of individualized intervention and providing technical support for the precise management of the entire MASLD CVD process, which is in line with multimodal AI The development direction of risk prediction models.

Despite its unlimited potential, we are still in the initial stage of transitioning from algorithms to routine clinical use. There is still a long way to go, and we will focus on how to better demonstrate prospective accuracy, conduct scientific research, and eliminate algorithm bias. We hope that artificial intelligence can help humans and machines work together, rather than replacing doctors. With the collaboration of the medical community, it can help manage today’s data volume and complexity, and immediately provide accurate and interpretable opinions to doctors. Healthcare professionals can focus their attention on things that truly make us human, such as empathy, judgment, and patient care skills. By joining forces with this vision, the medical community can also benefit a vast patient population around the world in the fight against MASLD and CVD.
